# The Probiotic *Phaeobacter inhibens* Provokes Hypertrophic Growth via Activation of the IGF-1/Akt Pathway during the Process of Metamorphosis of Greater Amberjack (*Seriola dumerili*, Risso 1810)

**DOI:** 10.3390/ani13132154

**Published:** 2023-06-29

**Authors:** Nikolas Panteli, Konstantinos Feidantsis, Maria Demertzioglou, Vasiliki Paralika, Stelios Karapanagiotis, Constantinos C. Mylonas, Konstantinos Ar. Kormas, Eleni Mente, Pavlos Makridis, Efthimia Antonopoulou

**Affiliations:** 1 Department of Zoology, School of Biology, Faculty of Sciences, Aristotle University of Thessaloniki, 54124 Thessaloniki, Greece; 2 Department of Fisheries & Aquaculture, University of Patras, 26504 Mesolonghi, Greece; 3 Department of Biology, University of Patras, 26504 Rio Achaias, Greece; 4 Galaxidi Marine Farm S.A., 33200 Galaxidi, Greece; 5 Institute of Marine Biology, Biotechnology and Aquaculture, Hellenic Center for Marine Research, P.O. Box 2214, 71003 Heraklion, Greece; 6 Department of Ichthyology and Aquatic Environment, School of Agricultural Sciences, University of Thessaly, 38446 Volos, Greece; 7 Agricultural Development Institute, University Research and Innovation Centre “IASON”, Argonafton & Filellinon, 38221 Volos, Greece; 8 Laboratory of Ichthyology-Culture and Pathology of Aquatic Animals, School of Veterinary Medicine, Aristotle University of Thessaloniki, 54124 Thessaloniki, Greece

**Keywords:** insulin-like growth factor type 1 receptor (IGF-1R), cellular signaling, hypertrophy, cell death, post-embryonic development, teleost, probiotics, aquaculture

## Abstract

**Simple Summary:**

Optimization of metamorphosis, a key stage in development where fish undergo several morphological and physiological changes, may assure the efficient mass production of greater amberjack (*Seriola dumerili*) juveniles. Application of probiotics as feed and/or water additive benefits nutrient utilization through modulation of the digestive enzymes. Therefore, following the addition of biofilters with the probiotic *Phaeobacter inhibens* in the rearing water throughout the early development of greater amberjack, crucial in metamorphosis, cellular pathways were investigated. The probiotic treatment increased the growth of greater amberjack, while hypertrophy was the main growth process in the metamorphosis. Additionally, the rearrangement of structures and tissues may have been facilitated through the observed induced cell death. The present findings are of great importance and may be applied in the aquaculture industry in order to enhance greater amberjack development and growth.

**Abstract:**

Metamorphosis entails hormonally regulated morphological and physiological changes requiring high energy levels. Probiotics as feed supplements generate ameliorative effects on host nutrient digestion and absorption. Thereby, the aim of the present research was to investigate the impact of the probiotic *Phaeobacter inhibens* as a water additive on cellular signaling pathways in the metamorphosis of greater amberjack (*Seriola dumerili*). Activation of insulin-like growth factor type 1 receptor (IGF-1R), protein kinase B (Akt), mitogen-activated protein kinases (MAPKs) and AMP-activated protein kinase (AMPK), induction of heat shock proteins (Hsps), and programmed cell death were assessed through SDS-Page/immunoblot analysis, while energy metabolism was determined through enzymatic activities. According to the results, greater amberjack reared in *P. inhibens*-enriched water entered the metamorphic phase with greater body length, while protein synthesis was triggered to facilitate the hypertrophic growth as indicated by IGF-1/Akt activation and AMPK inhibition. Contrarily, MAPKs levels were reduced, whereas variations in Hsps response were evident in the probiotic treatment. Apoptosis and autophagy were mobilized potentially for the structural remodeling processes. Furthermore, the elevated enzymatic activities of intermediary metabolism highlighted the excess energy demands of metamorphosis. Collectively, the present findings demonstrate that *P. inhibens* may reinforce nutrient utilization, thus leading greater amberjack to an advanced growth and developmental state.

## 1. Introduction

Metamorphosis, a crucial stage in teleost post-embryonic development, encompasses the irreversible, abrupt, and nonsexual ontogenetic transition from larval to juvenile, a sexually immature form of the adult stage [1]. Teleosts undergo several changes in morphological, physiological, and behavioral traits that precede the juvenile transition, such as maturation of internal organs, changes in gill mitochondria-rich cells, and conformation of the adult pigmentation pattern [1,2,3]. Metamorphic remodeling is governed by thyroid hormones, while other endocrine factors, such as cortisol, prolactin, and the growth hormone (GH)/insulin-like growth factor (IGF) system, are also involved in the regulation of several cellular and developmental processes [4,5].

The GH/IGF system, pivotal to growth regulation [6], is highly linked to the metamorphic success of teleosts [7,8,9]. The effects of GH on somatic growth are mediated by IGF-1, a polypeptide hormone mainly produced in the liver that exerts a plethora of physiological and developmental actions on target tissues in an endocrine manner [9,10,11]. Consequent to its GH-stimulated secretion in circulation, IGF-1 acts on cells by binding to the IGF-1 receptor (IGF-1R) [10,12,13]. The latter triggers IGF-1R autophosphorylation, thus leading to the activation of two intracellular downstream signaling pathways, the phosphatidylinositol 3-kinase/protein kinase B (PI3K/Akt) and the mitogen-activated protein kinase/extracellular signal-regulated kinase (MAPK/ERK) [13,14]. The PI3K/Akt transduces stimulatory signals for cellular differentiation [15], hypertrophy [16], and protein synthesis [17], fundamental factors in animals’ development and growth. Cellular uptake of amino acids and glucose is also modulated via the PI3K/Akt signaling to supply the required substrates and energy for growth-related anabolic processes [3,18]. In contrast, the proliferative function of IGF-1 is mediated via the MAPK/ERK pathway [14], which also comprises a critical regulator of cell terminal differentiation [19]. Additionally, MAPK/ERK signaling is involved in the up-regulation of heat shock proteins (Hsp) expression [20,21], which are implicated in several developmental stages [22,23], including induction and completion of metamorphosis [24,25].

Hormonally regulated programmed cell death also constitutes an essential component of normal developmental progression, facilitating ongoing tissue remodeling and functional reorganization [26]. During metamorphosis, apoptotic cell death is triggered for the degeneration of larva transient structures and the sculpting of adult-specific organs [27,28]. Furthermore, apoptosis functions as a quality control mechanism, ensuring the elimination of defective and surplus cells, as well as compensation for mispatterning [28,29]. Apoptosis is determined by the interplay between pro-apoptotic and anti-apoptotic signals and is implemented via the activation of the caspases cascade, which orchestrates the cleavage of cellular substrates [28,30]. Nevertheless, deletion and renovation of cells and tissues may also be facilitated via autophagy, an intracellular catabolic system that promotes the degradation and recycling of cytoplasmic constituents in the lysosome, thereby preserving energetic homeostasis in cells undergoing developmental procedures [29,31,32].

The initiation of metamorphosis is coordinated in response to miscellaneous physiological and environmental cues, including endocrine signaling, and changes in temperature and food availability [4,33,34]. The metamorphosing period is accompanied by high requirements for specific nutrients, vital in both growth and physiological processes such as hormone synthesis [35,36]. The application of probiotics as an environmentally friendly alternative to antibiotics has emerged, encouraging the development of a sustainable aquaculture industry [37]. Probiotics, defined as adjuncts to beneficial microbes or microbial components in the host’s environment or feed [37,38], have been reported to efficiently inhibit pathogens’ adhesion and enhance development, growth, feed utilization, and digestibility in several fish species [39,40,41,42]. Species of the marine *Roseobacter* clade, including *Phaeobacter inhibens*, which has recently been characterized as a “safe-to-use” probiotic [43,44], are common members of the prokaryotic communities in aquaculture systems [44,45,46] and effectively colonize a wide range of inorganic and organic surfaces [47]. Due to the ability to produce several unique bioactives, including the bactericidal antibiotic tropodithietic acid (TDA) [48], *P. inhibens* antagonistic activity and suppresses the growth of potential opportunistic pathogens for the reared populations, such as *Vibrio* and *Aeromonas* [45,49,50] with no adverse effects on the host [44]. Thereby, the aim of the present study was to explore the effects of the probiotic bacterium *P. inhibens* as a water additive on cellular and metabolic signaling pathways during the metamorphosis of greater amberjack (*Seriola dumerili*), a potential candidate species for Mediterranean aquaculture, the production of which faces several major bottlenecks related to nutrition and reproduction [51,52].

## 2. Materials and Methods

### 2.1. Application of Biofilters with Phaeobacter inhibens to Greater Amberjack Larval Rearing Conditions

The probiotic bacterial strain *Phaeobacter inhibens* [53] was provided by the Leibniz Institute DSMZ-German Collection of Microorganisms and Cell Cultures GmbH in the form of a lyophilized preparation. The strain was reactivated in 5 mL of Marine Broth (MB, Difco Laboratories, Detroit, MI, USA) by incubation at 22 °C for 7 days in the dark and stagnant conditions, and thereafter inoculated in 2 L conical flasks of MB for 7 days. Commercial aquaculture biofilter supporting medium of porous ceramic cylinders (Premium Filter Material) made from natural quartzite was selected for the growth of *P. inhibens* [54]. The ceramic cylinders were sterilized at 121 °C for 20 min.

*P. inhibens* liquid cultures were harvested from the 2 L conical flasks by centrifugation at 12,000× *g* at 18 °C to remove the liquid culture medium. Afterward, filtered autoclaved seawater was added to the probiotic cells, and they were transferred and equally divided into the 5 L glass beakers with the ceramic cylinders. Additional filtered autoclaved seawater was added until the cylinders were completely covered with seawater added with bacteria. The beakers were covered with aluminum foil and incubated in the dark at 22 °C for 7 days. The ceramic cylinders were thereafter rinsed with sterile seawater and placed in three plastic 10 L containers, which were covered with a 350 μm net to prevent greater amberjack larvae from entering the biofilter. They were separately and carefully placed inside three larval tanks. Aeration with an air stone was added to these simple biofilters constructed in this way to oxygenate the cylinders and create a continuous water exchange between the plastic container and the larval tank. In this way, cells of *P. inhibens* were spread to the whole water volume in the rearing tanks.

### 2.2. Rearing of Greater Amberjack Larvae

Equal amounts of fertilized greater amberjack eggs were stocked in six 2800 L volume cylindroconical tanks in a flow-through system at the hatchery facilities of Galaxidi Marine Farm S.A., Greece. Borehole seawater was sand-filtered, aerated, and UV-treated prior to its use in the rearing system. The biofilters with the probiotic *P. inhibens* had been submerged in three tanks three days prior to the transfer of the amberjack eggs. The desired water temperature (>23 °C) was achieved using heat pumps. After hatching, the larval density was approximately 56 larvae/L. During the experimental period, the temperature and pH remained stable at 23–24 °C and 8, respectively. The dissolved oxygen saturation level was 85–95%. Water renewal was adjusted to 10% tank volume/h during egg incubation, was reduced to 5% tank volume/h on the day of hatching, and thereafter was increased gradually from 6 days post-hatching (dph) onwards up to 30% tank volume/h. Lights were turned on at 3 dph at 1000 lux intensity, decreased to 800 lux at 11 dph, 700 lux at 19 dph, and 500 lux at 23 dph. The live feed diet consisted of Rotifers (*Brachionus* sp.) from 3 to 22 dph, newly hatched *Artemia* nauplii from 12 to 33 dph, and enriched *Artemia* metanauplii from 18 dph up to the end of the trial. Formulated feed (Caviar Nature, BernAqua, Olen, Belgium) was used from 23 dph onwards.

Throughout the experimental trial, total length was used as the main morphometric index for the assessment of greater amberjack developmental progress [55,56]. On day 33, post-hatching during the process of metamorphosis and, subsequently, to the completion of notochord flexion, larvae from both experimental and control groups were sampled. From each of the 3 tanks, two biological replicates were collected (N = 2 + 2 + 2 = 6). Fish were captured, placed in 3 mL vials, and immediately frozen using liquid nitrogen. Samples were later transferred to −80 °C until further analyses.

### 2.3. SDS/PAGE and Immunoblot Analysis

The preparation of samples for SDS-PAGE, quantification of caspases and ubiquitinated proteins, and the immunoblot analysis are based on well-established protocols [57]. Specifically, frozen tissues were immediately homogenized in 3 mL g^−1^ of cold lysis buffer (20 mM β-glycerophosphate, 50 mM NaF, 2 mM EDTA, 20 mM Hepes, 0.2 mM Na_3_VO_4_, 10 mM benzamidine, pH 7, 200 μM leupeptin, 10 μΜ trans-epoxy succinyl-Lleucylamido-(4-guanidino)butane, 5 mM dithiotheitol, 300 μΜ phenyl methyl-sulfonyl fluoride (PMSF), 50 μg mL^−1^ pepstatin, 1% *v*/*v* Triton X-100), and extracted on ice for 30 min. Samples were centrifuged (10,000× *g*, 10 min, 4 °C), and the supernatant was boiled with 0.33 volumes of SDS/PAGE sample buffer (330 mM Tris-HCl, 13% *v*/*v* glycerol, 133 mM DTT, 10% *w*/*v* SDS, 0.2% *w*/*v* bromophenol blue). For the determination of LC3 II/I ratio and SQSTM1/p62 levels, samples were lysed in a buffer containing 150 mM NaCl, 20 mM Hepes, 5 mM DTT, 0.3 mM PMSF, 0.2 mM leupeptin, 0.01 mM E64 and 1% Triton X-100. Lysates were incubated on ice for 30 min and then centrifuged at 4 °C, for 5 min at 3000× *g*. Protein concentration was determined by using the BioRad protein assay (Bio-Rad Protein Assay Kit I, 5000001, BioRad, Hercules, CA, USA).

For the SDS-PAGE, equivalent amounts of proteins (50 μg) from samples of 5 individual batches of each developmental stage were separated either on 10% and 0.275% or 15% and 0.33% (*w*/*v*) acrylamide and bisacrylamide, followed by electrophoretic transfer onto membranes of nitrocellulose (0.45 μm, Schleicher and Schuell, Keene, NH, USA).

The resulting membranes were subjected to overnight incubation with: polyclonal rabbit anti-bcl2 (7973, Abcam, Cambridge, UK), polyclonal rabbit anti-bax (B-9) (7480, Santa Cruz Biotechnology, Dallas, TX, USA), monoclonal mouse anti-HSP70 (H5147, Sigma, St. Louis, MO, USA), monoclonal mouse anti-HSP90 (H1775, Sigma), anti-HSP60 (12165, Cell Signaling, Beverly, MA, USA), monoclonal mouse anti-phospho-SAPK-JNK (9255, Cell Signaling, Beverly, MA, USA), polyclonal rabbit anti-phospho-p38 MAP kinase (9211, Cell Signaling, Beverly, MA, USA), monoclonal rabbit anti-phospho p44/42 MAPK (4376, Cell Signaling, Beverly, MA, USA), polyclonal rabbit anti-SAPK-JNK (9252, Cell Signaling, Beverly, MA, USA), polyclonal rabbit anti-p44/42 MAPK (4695, Cell Signaling, Beverly, MA, USA), polyclonal rabbit anti-p38 MAPK (9212, Cell Signaling, Beverly, MA, USA), monoclonal rabbit anti-LC3B (3868, Cell Signaling, Beverly, MA, USA), polyclonal rabbit anti-p62/SQSTM1 (5114, Cell Signaling, Beverly, MA, USA), monoclonal rabbit anti-phospho AMPK (2535, Cell Signaling, Beverly, MA, USA), monoclonal rabbit anti-AMPK (5831, Cell Signaling, Beverly, MA, USA), anti-phospho-IGF-1R (3918, Cell Signaling, Beverly, MA, USA), anti-IGF-1R (9750, Cell Signaling, Beverly, MA, USA) and anti-phospho-Akt (9271, Cell Signaling, Beverly, MA, USA), anti-Akt (9272, Cell Signaling, Beverly, MA, USA). Quality transfer and protein loading Western blot were assured by Ponceau stain and actin (anti-β actin 3700, Cell Signaling, Beverly, MA, USA). Each of the examined proteins was normalized with its respective β-actin.

Concerning cleaved caspases and ubiquitination levels, protein samples were immunoblotted with a dot blot apparatus employment [58], and membranes were overnight incubated with monoclonal rabbit anti-cleaved caspase antibody (8698, Cell Signaling) and monoclonal mouse anti-ubiquitin conjugate (3936, Cell Signaling). Bands and blots were detected by enhanced chemiluminescence, while quantification was applied through laser-scanning densitometry (GelPro 4.0 Analyzer Software, GraphPad, San Diego, CA, USA).

### 2.4. Determination of Intermediate Metabolism Enzyme Activities

Activities of lactate dehydrogenase (L-LDH; E.C. 1.1.1.27.), citrate synthase (CS; E.C. 4.1.3.7.), and 3-hydroxyacyl CoA dehydrogenase (HOAD; 1.1.1.35) were estimated in samples according to well-established techniques [59,60,61,62]. For the analysis of L-LDH and HOAD activities, samples were homogenized in a buffer containing 150 mM imidazole, 1 mM EDTA, 5 mM dithiothreitol (DTT), and 1% Triton X-100, pH 7.4. For CS activity, tissue samples were homogenized in a buffer containing 20 mM HEPES, and 1 mM EDTA, with 1% Triton X-100, pH 7.4. To avoid loss of enzyme activity during sample preparation, procedures were performed on ice. Before analysis, homogenates were centrifuged at 13,000× *g* for 10 min at 4 °C. Maximum activity levels were determined spectrophotometrically at 18 °C. L-LDH and HOAD enzyme activities were measured following the oxidation of NADH at 340 nm (mM extinction coefficient = 6.22), and CS enzyme activities were determined based on the reaction of free coenzyme A with DTNB (5.5 V dithio-bis (2- nitrobenzoic acid) at 412 nm (mM extinction coefficient = 13.6). L-LDH was assayed in a medium containing 0.15 mM NADH, 1 mM KCN, and 50 mM imidazole, pH 7.4. The reaction was initiated by adding 1 mM pyruvate. CS was assayed in a medium containing 0.4 mM acetyl CoA, 0.25 mM DTNB and 75 mM Tris buffer, pH 8.0. The reaction was initiated by adding 0.5 mM oxaloacetate (OAA). 3-hydroxyacyl CoA dehydrogenase was assayed in a medium containing 0.15 NADH, 1 mM KCN, 1 mM EDTA, 50 mM Imidazole, pH 7.4. The reaction was initiated by the addition of 2.0 mM acetoacetate. Enzyme activities are expressed as micromoles of substrate min/mg protein. Protein concentration in supernatants was determined by using the BioRad protein assay (Bio-Rad Protein Assay Kit I, 5000001, BioRad, Hercules, CA, USA).

### 2.5. Microbiological Analysis of Rearing Water

In order to examine the effect of *P. inhibens* on bacterial load in the water, serial ten-fold dilutions of seawater from the rearing tanks from each sampling point were plated on 90 mm Petri dishes of Marine Agar (Difco Laboratories, Detroit, MI, USA) and thiosulfate-citrate-bile salts-sucrose agar (TCBS). The Petri dishes were thereafter incubated at room temperature (20 °C) for seven days in the dark, and the bacterial colonies were counted 1, 2, and 7 days after sampling.

### 2.6. Statistical Analysis

Protein expression data were tested for normality of distribution via the Shapiro–Wilk test, while homogeneity of variance was assessed using Levene’s Test for Equality of Variances. Changes in biochemical responses were tested for significance at the 5% level by using a one-way Analysis of variance (ANOVA) (GraphPad Instat 3.0). Values are presented as means ± S.D.

Principal components analysis (PCA) was conducted in the R package FactoMineR to assess patterns of possibly correlated variables. Moreover, simple linear correlation (Pearson’s test) analysis was employed for the estimation of significant correlations (at a 5% level) between the biochemical data obtained in the present study (GraphPad Prism 5.0).

## 3. Results

**Figure 1:**
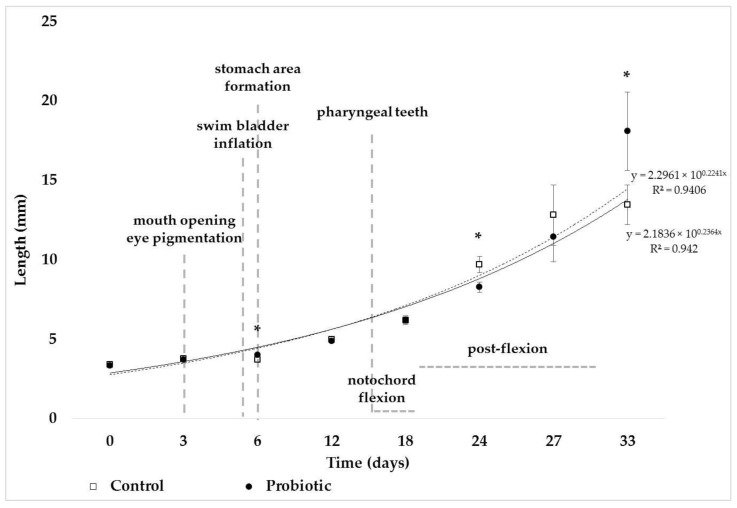
Temporal changes in total body length (mean values, N = 12) of greater amberjack (*Seriola dumerili*) from hatching day (D0) until metamorphosis (D33) in the absence and presence of *Phaeobacter inhibens* in the rearing water. Asterisks (*) depict significant differences (*p* < 0.05) between the treatments.

According to the measured total body length, the larval notochord flexion was completed by the end of the experimental trials. Changes in the total body length of greater amberjack from hatching day until metamorphosis (33 dph) are depicted in Figure 1. Growth in total body length was significantly higher (*p* < 0.05) in greater amberjack reared in the *P. inhibens*-enriched water at 6 dph and 33 dph. In contrast, lower total body length (*p* < 0.05) was evident in greater amberjack of the *P. inhibens* treatment at 24 dph compared to the control.

**Figure 2:**
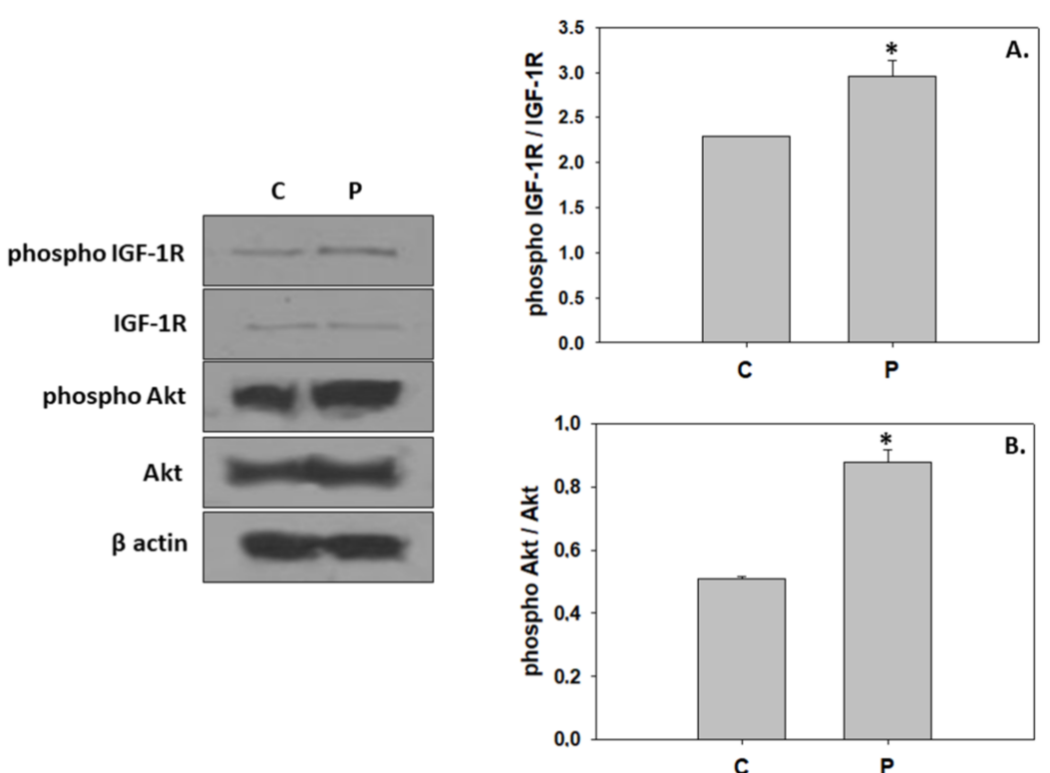
Levels of phospho IGF-1R/IGF-1R (**A**) and phospho Akt/Akt (**B**) (mean ± std) in the absence (C) and presence (P) of *Phaeobacter inhibens* in the rearing water of greater amberjack (*Seriola dumerili*). The quantitative histograms show the changes in the above-mentioned indicators under C and P treatment, normalized with β-actin. Representative blots are shown. N = 6 preparations from different animals. Significant differences (*p* < 0.05) are presented as *.

**Figure 3:**
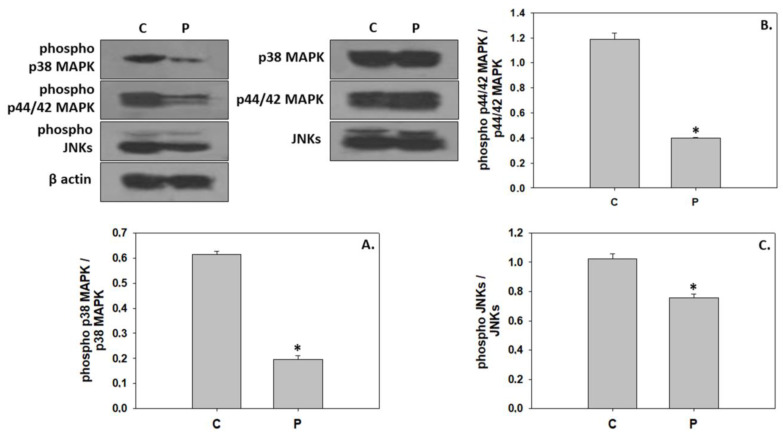
Levels of phospho p38 MAPK/p38 MAPK (**A**), phospho p44/42 MAPK/p44/42 MAPK (**B**), and phospho JNKs/JNKs (**C**) levels (mean ± std) in the absence (C) and presence (P) of *Phaeobacter inhibens* in the rearing water of greater amberjack (*Seriola dumerili*). The quantitative histograms show the changes in the above-mentioned indicators under C and P treatment, normalized with β-actin. Representative blots are shown. N = 6 preparations from different animals. Significant differences (*p* < 0.05) are presented as *.

**Figure 4:**
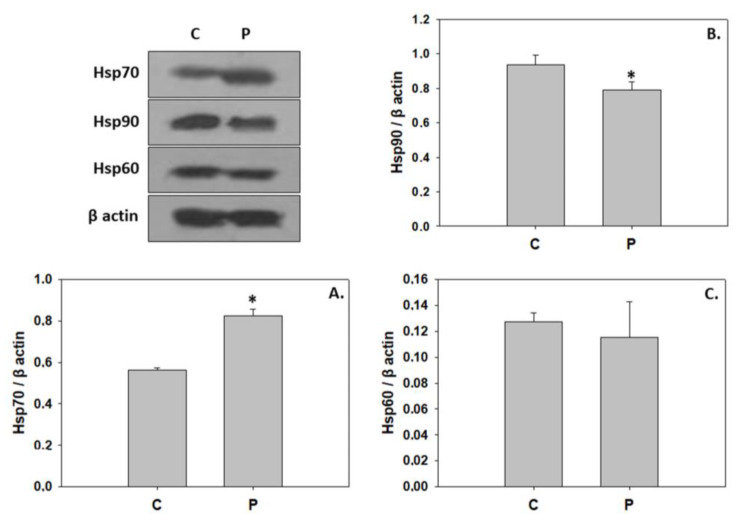
Levels of Hsp70 (**A**), Hsp90 (**B**), and Hsp60 (**C**) induction (mean ± std) in the absence (C) and presence (P) of *Phaeobacter inhibens* in the rearing water of greater amberjack (*Seriola dumerili*). The quantitative histograms show the changes in the above-mentioned indicators under C and P treatment, normalized with β-actin. Representative blots are shown. N = 6 preparations from different animals. Significant differences (*p* < 0.05) are presented as *.

The probiotic *P. inhibens* as a water additive displayed similar effects on the phosphorylation ratios of IGF-1R and Akt, resulting in a significant activation (Figure 2). In contrast, probiotic-enriched water provoked a significant reduction in the phosphorylation ratio of all three MAPKs examined in the present study (Figure 3). The addition of the probiotic in the rearing water did not affect Hsp60 expression in 33 dph greater amberjack. However, probiotic addition significantly reduced Hsp90 levels, whereas Hsp70 levels were significantly increased (Figure 4).

**Figure 5:**
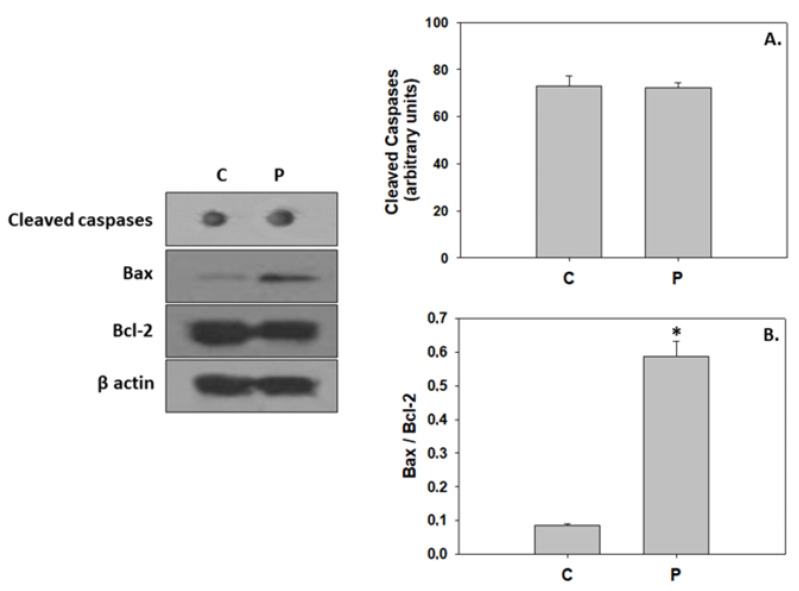
Cleaved caspases (**A**) and Bax/Bcl-2 ratio (**B**) levels (mean ± std) in the absence (C) and presence (P) of *Phaeobacter inhibens* in the rearing water of greater amberjack (*Seriola dumerili*). The quantitative histograms show the changes in the above-mentioned indicators under C and P treatment, normalized with β-actin. Representative dots and blots are shown. N = 6 preparations from different animals. Significant differences (*p* < 0.05) are presented as *.

**Figure 6:**
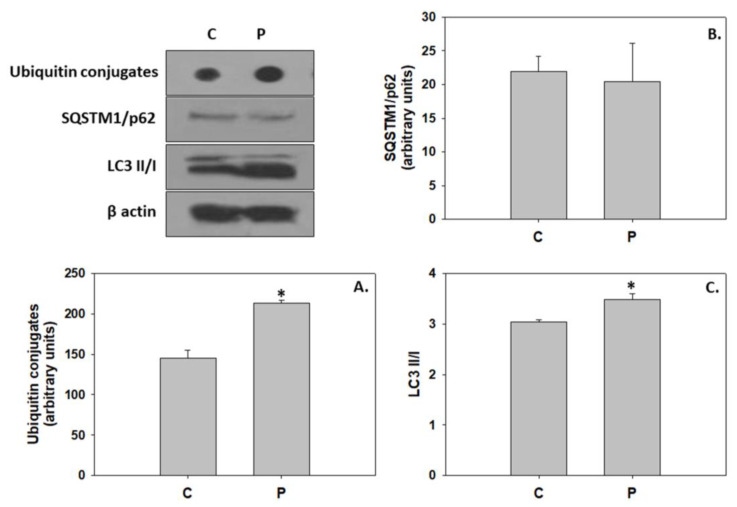
Ubiquitin conjugates (**A**), SQSTM1/p62 (**B**), and LC3 II/I (**C**) levels (mean ± std) in the absence (C) and presence (P) of *Phaeobacter inhibens* in the rearing water of greater amberjack (*Seriola dumerili*). The quantitative histograms show the changes in the above-mentioned indicators under C and P treatment, normalized with β-actin. Representative dots and blots are shown. N = 6 preparations from different animals. Significant differences (*p* < 0.05) are presented as *.

**Figure 7:**
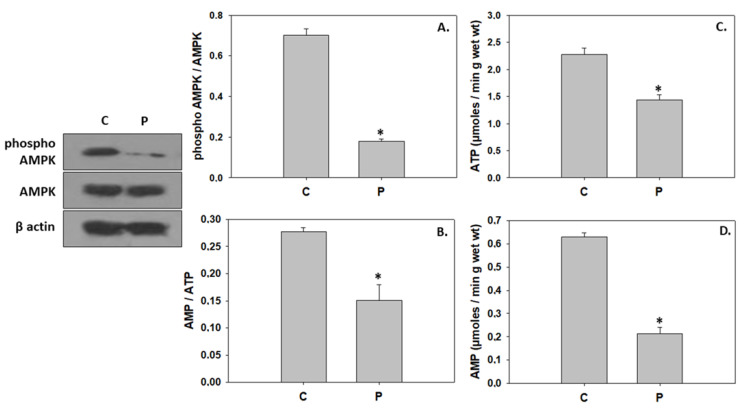
Levels of phospho AMPK/AMPK (**A**), AMP/ATP (**B**), ATP (**C**), and AMP (**D**) (mean ± std) in the absence (C) and presence (P) of *Phaeobacter inhibens* in the rearing water of greater amberjack (*Seriola dumerili*). The quantitative histograms show the changes in the above-mentioned indicators under C and P treatment, normalized with β-actin. Representative blots are shown. N = 6 preparations from different animals. Significant differences (*p* < 0.05) are presented as *.

The addition of the probiotic did not alter caspase levels compared to the control group. However, the probiotic significantly increased the Bax/Bcl-2 compared to the control (Figure 5). Regarding autophagic indicators, while the addition of the probiotic resulted in a statistically significant increase of ubiquitin conjugates levels and LC3 II/I ratio, no changes were observed in the SQSTM1/p62 levels (Figure 6). AMPK phosphorylation, the AMP/ATP ratio, and ATP and AMP levels were significantly reduced in the probiotic-enriched water compared to the control (Figure 7).

**Figure 8:**
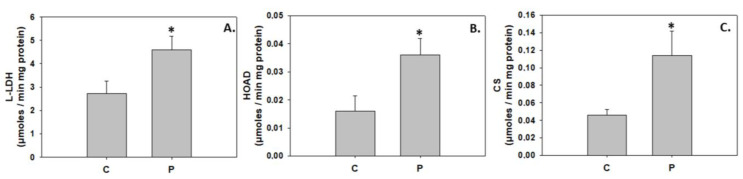
Levels of L-LDH (**A**), HOAD (**B**), and CS (**C**) activity (mean ± std) in the absence (C) and presence (P) of *Phaeobacter inhibens* in the rearing water of greater amberjack (*Seriola dumerili*). Representative blots are shown. N = 6 preparations from different animals. Significant differences (*p* < 0.05) are presented as *.

On the other hand, L-LDH, HOAD, and CS activity levels were significantly increased in the probiotic treatment (Figure 8).

**Figure 9:**
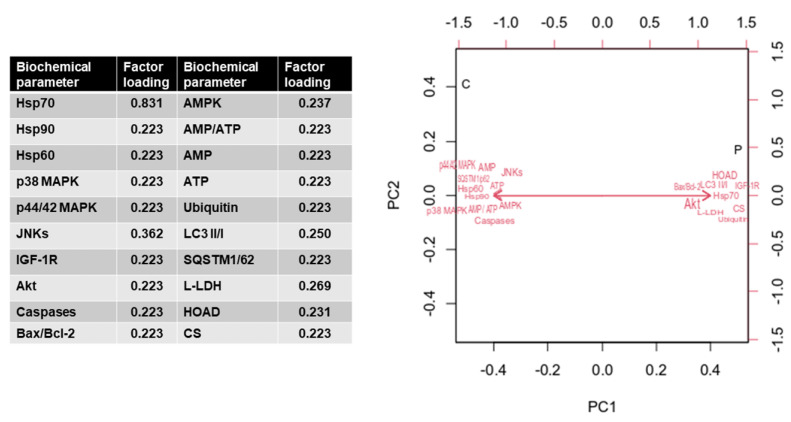
An analytical table of the contribution of biochemical parameters was studied according to factor loadings and variable correlations with each of the first two principal components (PCs) in the multivariate analysis. The PCA was generated from the complete biochemical and physiological dataset. Parameters with red vector arrows were included as predictors in constructing the PCA.

According to the Principal Components Analysis (PCA), PC1 explained 31.62% of the variance (Figure 9). The biochemical variables that were positively correlated with PC1 scores were Hsp70, IGF-1R, Akt, Bax/Bcl-2, Ubiquitin, LC3 II/I, L-LDH, HOAD, and CS. In contrast, Hsp90, Hsp60, p38 MAPK, p44/42 MAPK, JNKs, caspases, AMPK, AMP/ATP, AMP, ATP, and SQSTM1/p62 were negatively correlated with PC1. Regarding PC2, which explained 27.75% of the variance, the scores that were positively correlated to this principal component were JNKs, IGF-1R, Akt, Bax/Bcl-2, AMP, ubiquitin, LC3 II/I, and L-LDH. In contrast, Hsp70, Hsp90, Hsp60, p38 MAPK, p44/42 MAPK, caspases, AMPK, AMP/ATP, ATP, SQSTM1/p62, HOAD, and CS were negatively correlated to PC2. The cumulative value of PC1 and PC2 was 66.09%.

The bacterial load in the rearing water, as determined by the total counts in colony-forming units (CFU) per mL, was not significantly modified among the *P. inhibens*-enriched tanks and the control tanks (*p* > 0.05) (Supplementary Material Figure S1). The number of presumptive *Vibrios*, as determined by CFU counts in TCBS agar, was similar in both treatments (*p* > 0.05), despite the higher numbers in the control tanks (Supplementary Material Figure S1).

## 4. Discussion

Growth in fish is highly reflected by the growth of skeletal muscle, the most abundant tissue and main contributor to total body mass [63]. In contrast to higher vertebrates, post-hatching muscle growth in teleost species is implemented by both hypertrophy (enlargement of existing muscle fibers) and hyperplasia (the genesis of new myofibers) [64]. During a teleost life span, the equilibrium in dominance among the two aforementioned processes is differentiated in a species-specific manner, where hypertrophy seems to constitute the main growth mechanism towards the later developmental stages of juvenile and adult, as has been previously demonstrated in Gilthead seabream, *Sparus aurata*, and white seabass, *Atractoscion nobilis* [63,65].

### 4.1. Induction of Hypertrophic Growth via Activation of IGF/Akt Pathway

According to the present results, administration of the probiotic *P. inhibens* in the rearing water promoted the activation of both IGF-1R and Akt, integral mediators of hypertrophic growth [16]. Nutritional regulation of the IGF-1/PI3K/Akt pathway has been previously reported in the skeletal muscle of fine flounder (*Paralichthys adspersus*) [66] and rainbow trout (*Oncorhynchus mykiss*) [67,68], where a dietary trial of fasting and refeeding led to PI3K/Akt inactivation and activation, respectively. The ameliorative effects of probiotics in host nutrition have been extensively documented in several teleost species. For instance, dietary supplementation of probiotics enhanced the activities of digestive enzymes, such as lipase, amylase, alkaline phosphatase, pepsin, trypsin, and chymotrypsin, and improved the growth performance in Caspian white fish (*Rutilus frisii kutum*) [69], California halibut (*P. californicus*) [70], zebrafish (*Danio rerio*) [71], European seabass (*Dicentrarchus labrax*) [72] (and gilthead sea bream [73]. In addition, probiotics may beneficially modulate gut microbiota composition and function, and ameliorate microbial balance, which may further improve the physiological processes in the digestive system (reviewed in [69,74]). Thus, the development of greater amberjack in rearing water supplemented with *P. inhibens* may have favored the digestion and absorption of nutrients such as proteins and, in consequence, altered cellular metabolic pathways.

Specifically, Akt activation observed herein, possibly due to digestion strengthening, may mediate the nutrient- and IGF-induced signaling for stimulation of protein synthesis via phosphorylation of the target of rapamycin (TOR), a downstream component of the PI3K/Akt pathway (reviewed in [75,76]). Induction of protein synthesis through the TOR pathway has been previously demonstrated in juvenile Jian carp (*C. carpio* var. Jian) in response to fish meal substitution with yeast hydrolysate [77]. In contrast, dietary supplementation of cottonseed meal protein hydrolysate at high levels repressed protein synthesis due to inhibition of the TOR signaling pathway via activation of AMPK [78]. The AMP-activated protein kinase is an energy-status sensor, which is activated responsively to changes in the intracellular AMP/ATP ratio and, more precisely, under a low cellular energetic regime in order to suppress energy-consuming processes such as protein synthesis and to stimulate catabolic pathways for ATP regeneration in the cell [76,79]. Thereby, the apparent low ratio of AMP/ATP and the inhibition of AMPK observed herein in greater amberjack reared in the probiotic-enriched water further confirms IGF/Akt/TOR-stimulated protein synthesis, which may have been recruited for the hypertrophic growth during metamorphosis. The present finding is consistent with previous studies that indicated the prevailing hypertrophy in postnatal growth, including the metamorphosing stage, in several teleosts [55,80,81]. However, in addition to the rapid growth of greater amberjack, the IGF/Akt-mediated hypertrophy may be implicated in the metamorphic remodeling and reorganization of miscellaneous larvae tissues and structures, such as the gastrointestinal tract as observed in other species [82], in order to functionally adapt to the juvenile form.

### 4.2. Inhibition of Downstream Components in the IGF/MAPK Signaling

In contrast to the IGF/Akt activation, *P. inhibens* as a water additive suppressed components of the MAPK signaling pathway during greater amberjack metamorphosis. Activation of the MAPK pathway via IGF-1 binding to the IGF-1R signals for cell proliferation and hyperplastic growth [15]. Proliferative events have previously been demonstrated to be crucial to the structural and functional development of tissues and organs during teleosts’ metamorphosis [83,84]. In regard to greater amberjack, Panteli et al. [85] indicated p38 MAPK, p44/42 MAPK, and JNK activation in the metamorphosing stage, thus indicating a potential involvement in organogenesis and muscle growth. Herein, *P. inhibens* may have acted as an inhibitor of the hyperplastic effect as observed by p38 MAPK, p44/42 MAPK, and JNK inactivation compared to the greater amberjack of the control group. In line with the present results, probiotic-supplemented diets increased the growth rate of Malaysian Mahseer (*Tor tambroides*) juveniles mainly through the prevalence of hypertrophic growth process in muscle fibers rather than hyperplasia [86].

The modification in MAPKs activation observed herein may be attributed to the evident differences in the greater amberjack length among the two groups. Specifically, according to the total length, the greater amberjack reared in the *P. inhibens*-enriched water may have initiated the stage of metamorphosis earlier in comparison to the control group. The relative contribution of hyperplasia in the growth procedure decreases with standard fish length, as reported by Zimmerman and Lowery [87]. Therefore, the apparent decrease in p38 MAPK, p44/42 MAPK, and JNK activation may indicate that *P. inhibens* favored a more rapid growth, potentially leading greater amberjack to advanced development, where the hyperplastic-facilitated procedures were mostly completed. Furthermore, hypertrophy and hyperplasia function in a cyclic phase pattern during teleosts’ early ontogeny [80,87], which reinforces the latter hypothesis. In this context, Weston et al. [88] suggested that p38 signaling is active early in myogenesis in order to prevent myocyte premature progression and is subsequently suppressed during the elongation, polarization, aggregation, and fusion of the differentiated cells. However, it is worth mentioning that fish species of large final body size, such as greater amberjack, continue to rely to an extent on hyperplasia for muscle growth in the adult phase [63].

Furthermore, consequent to the decrease in MAPKs phosphorylation, the expression level of Hsp90 was downregulated in the greater amberjack reared under the *P. inhibens* regime. As previously mentioned, MAPKs are regulators involved in Hsps induction [20,21]. According to previous studies, the metamorphosis of greater amberjack [85] and flatfish Senegalese sole (*Solea senegalensis* Kaup) [23] was accompanied by a decrease in Hsp90 expression levels. However, *P. inhibens* as a water additive induced Hsp70 expression in this instance, which was also evident in gilthead seabream following probiotics administration [89]. On the contrary, decreased Hsp70 levels were observed in teleost species under probiotic treatment [39,90,91]. Changes in Hsps induction due to probiotics vary according to miscellaneous factors such as fish species, development stage, bacterial strain and dose, supplementation mode, and rearing duration and conditions [90]. Molecular chaperones are involved in the regulation of signal-transduction pathways [23], and thus, the recruitment of such proteins is essential in several developmental stages, including metamorphosis for tissue resorption and rearrangement [92]. Upon entering the metamorphic phase, silver sea bream (*Sparus sarba*) exhibited induction of both Hsp70 and Hsp90, therefore indicating involvement in the larvae to juvenile transition processes [93]. However, Chung-Davidson et al. [94] demonstrated that Hsp90 inhibition facilitates the degeneration stage of the gall bladder during the liver metamorphosis of sea lamprey. Therefore, both up-regulation and down-regulation of Hsps due to the addition of *P. inhibens* may be deemed crucial in proper greater amberjack metamorphosis and accelerated growth, and hence, further investigations are required to clarify their distinct role in the organism’s development.

### 4.3. P. inhibens-Induced Developmental Cell Death

The commencement of metamorphosis in vertebrates is associated with the recruitment of extensive programmed cell death processes in order to facilitate the deletion of larval cells and tissues for the structural, morphological, and functional remodeling of the juveniles (reviewed in [26,30]). For instance, during the initial stage of the metamorphosis of the Japanese flounder (*Paralichthys olivaceus*), pro-apoptotic signals were evident in the frontal bone, and ethmoid and trabecular cartilage, which may have subsequently contributed to eye migration [95,96]. In addition, Gao et al. [97] demonstrated that the autophagic machinery facilitates eye migration in the aforementioned species through cell death in the orbital tissue. The addition of *P. inhibens* in the rearing water resulted in the induction of both apoptosis and autophagy in greater amberjack, as observed by the increased Bax/Bcl-2 and LC3 II/I ratios. The action of induced apoptosis was demonstrated in teleost cell lines following the addition of cytoplasmic extracts obtained from the probiotic *Lactobacillus delbrüeckii* subsp. *lactis* [98]. As indicated in the present results, probiotic treatment induced autophagy in zebrafish follicles, as observed by the increased autophagosomes and LC3-II protein levels, which may participate in ubiquitinated protein degradation [99]. Mobilization of apoptotic cell death observed herein may indicate the increased structural processes due to the potential accelerated growth. In specific, under the *P. inhibens* regime, greater amberjack seems to exhibit advanced developmental progress compared to the control.

However, probiotic-induced autophagy may subserve a dual role in greater amberjack metamorphosis. In addition to the aforementioned role in larval to juvenile reconstruction, autophagy may have also been employed to secure energy and nutrients required for the energetically demanding processes of metamorphosis and growth [29,31,100]. The latter is further supported by the increased activities of intermediary metabolism enzymes in the greater amberjack of the *P. inhibens* treatment despite the AMPK inhibition. In line with the above, probiotic-enhanced nutrient metabolism and growth performance was similarly suggested by Yi et al. [101], who demonstrated an up-regulation of genes related to carbohydrate metabolism and growth, including the IGF-1 gene, in zebrafish fed a diet supplemented with the potential probiotic *Chromobacterium aquaticum*. Skeletal muscle growth depends on protein anabolism [102], an energy-consuming procedure, and thus additional energy may be required by the greater amberjack reared under the *P. inhibens* regime, which also displayed higher body length.

## 5. Conclusions

**Figure 10:**
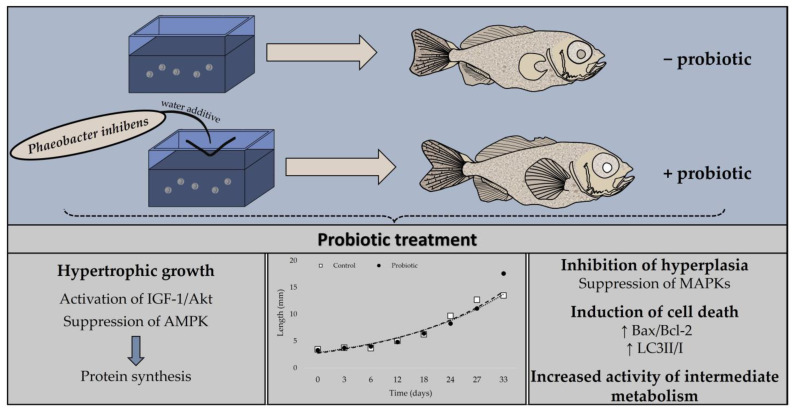
Summarized model of the modified biochemical indicators under the probiotic treatment during the metamorphosis of greater amberjack (*Seriola dumerili*).

Administration of probiotic *Phaeobacter inhibens* as water additive exerted a contrary indirect regulatory role on IGF-I/Akt and IGF-I/MAPK signaling in the metamorphosis of greater amberjack. Specifically, the potentially higher nutrient supply due to the *P. inhibens*-enhanced digestion triggered the activation of the IGF-I/Akt pathway, while downstream components of the IGF-I/MAPK, including p38 MAPK, p44/42 MAPK, and JNK exhibited inhibition (Figure 10). Moreover, inhibition of AMPK indicates that anabolic processes, such as protein synthesis, are active and facilitate hypertrophic growth. Induced programmed cell death may have been recruited in the probiotic treatment for the deletion and renovation of cells and tissues. According to the present results, it can be assumed that under the influence of the probiotic treatment, an apparent increase in the rate of development and muscle hyperplasia occurred earlier, thus leading to the subsequently enhanced hypertrophy observed. It should be emphasized that larval muscle hyperplasia and hypertrophy may affect the post-metamorphosis muscle cellularity, and the relative contributions of these two growth mechanisms exert long-term effects on muscle cellularity and growth of reared fish [103,104]. In conclusion, *P. inhibens* as a water additive seems to exert ameliorative effects in the development of reared greater amberjack. However, both developmental processes and responses to probiotics depend highly on species; thus, more species-oriented research is required to further explore the application of *P. inhibens* in the aquaculture industry, as well as the long-term change effects in hyperplasia and hypertrophy.

## Data Availability

The datasets generated and/or analyzed during the current study are not publicly available because, due to the nature of this research, accompanying data to the ones presented herein remain unpublished to date, but are available from the corresponding author on reasonable request.

## Supplementary Materials

The following supporting information can be downloaded at: https://www.mdpi.com/article/10.3390/ani13132154/s1, Figure S1: Total bacterial load and presumptive *Vibrios* in seawater from the rearing tanks as determined by counting of colony forming units (CFU) in Marine agar and in TCBS agar, respectively.
