# Supplementary material for: The Probiotic Phaeobacter inhibens Provokes Hypertrophic Growth via Activation of the IGF-1/Akt Pathway during the Process of Metamorphosis of Greater Amberjack (Seriola dumerili, Risso 1810)
Source: Animals (Basel). 2023 Jun 29;13(13):2154. doi: 10.3390/ani13132154 (PMC10340036; doi:10.3390/ani13132154)

Figure S1. Total bacterial load and presumptive *Vibrios* in seawater from the rearing tanks as determined by counting of colony forming units (CFU) in Marine agar and in TCBS agar, respectively.

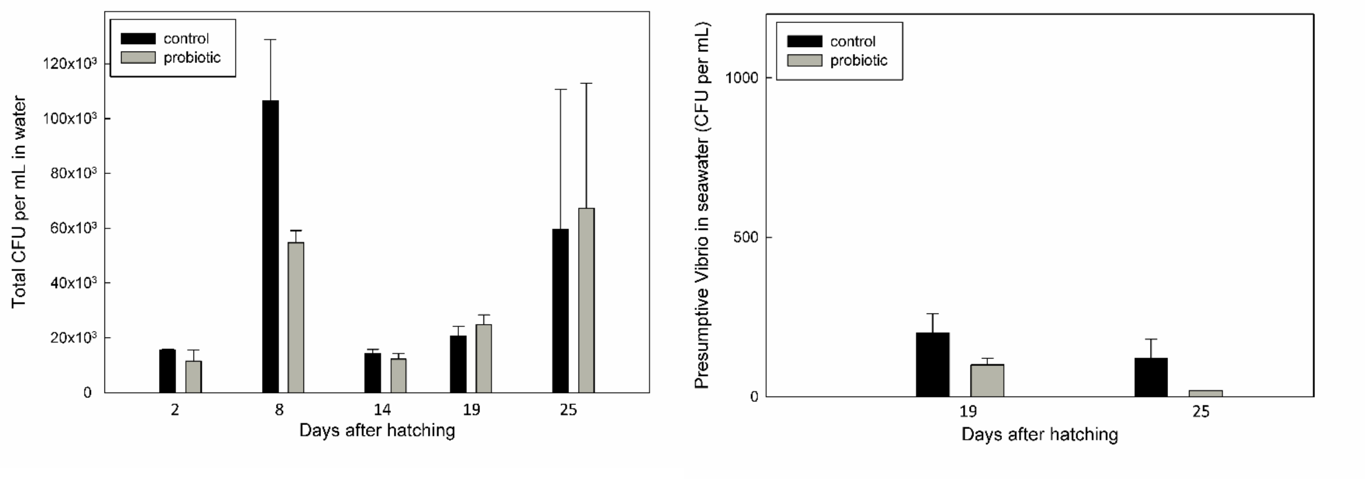

Supplement: Supplementary file 1 [file animals-13-02154-s001.zip › animals-2273653-supplementary.pdf]
